# Covering surgical instruments with single- or double-layer drape pending surgery: an experimental study in a perioperative setting

**DOI:** 10.1177/1757177420973753

**Published:** 2020-12-08

**Authors:** Maria Qvistgaard, Sofia Almerud-Österberg, Jenny Lovebo

**Affiliations:** Health and Caring Sciences, Linnaeus University, Växjö, Sweden

**Keywords:** Colony forming unit, perioperative care, surgical site infection, surgical instrument

## Abstract

**Background:**

Surgical site infections (SSI) constitute a severe threat to surgery patients. The surgical environment must be as free of contaminating microorganisms as possible. Using sterile surgical instruments while performing surgery is an absolute necessity for ensuring quality of care in perioperative settings.

**Aim:**

To compare bacterial contamination of agar plates after 15 h on set surgical instrument tables covered with a single- or double-layer drape.

**Methods:**

An experimental design was used consisting of set instrument tables with six agar plates on each table: four instrument tables were covered with a single-layer drape and four instrument tables were covered with a double-layer drape. This set-up was repeated on nine occasions during the period of data collection, making 76 set instrument tables in total. As a control, one instrument table was uncovered on four of those occasions.

**Results:**

The double-layer drape cover showed a significantly (*P* = 0.03) lower number of colony forming units (CFU) per agar plate than the single-layer drape covering. As expected, the uncovered instrument tables were highly contaminated.

**Discussion:**

Our results indicate that it is good practice to cover instruments properly with at least a single-layer drape before a surgical procedure. If there is difficulty achieving optimal conditions while setting the instrument tables (e.g. positioning the patient for general anaesthesia), it is a better option to set the instrument tables earlier and cover them with a double-layer drape. These precautions will help protect the patient from harm and unnecessary SSI by lowering microbiological burden, a key factor in developing SSI.

## Background

Surgical site infections (SSI) constitute a severe threat to surgery patients. Prevalence of SSI depends on several factors, but approximately 5% of all surgical procedures lead to minor or major infections (Bagnall et al., 2009). Until recently, there were options for treatment of infections, but these options are decreasing due to antibiotic resistance (Berrios-Torres et al., 2017). In an optimal perioperative setting, only sterile surgical instruments are used; however, the longer surgical instruments are exposed to air, the more likely they are to be contaminated (Dalstrom et al., 2008, Saito et al., 2014). Perioperative care needs to investigate every way of keeping the operating room (OR) environment, and especially the surgical instruments, as contamination-free as possible in order to ensure patient safety with regard to SSIs (Meeks et al., 2011). OR nurses in the surgical team, in particular, are responsible for minimising the environmental risks of SSI (Qvistgaard et al., 2019), so properly trained and evaluated registered nurses (RN) increase patient safety and quality of care. Therefore, evidence-based perioperative care is needed for safe care. However, RNs are an underused resource when it comes to minimising the risks of SSI (Smeds-Alenius et al., 2016). Patient safety with respect to SSI is an important concern due to its economic burden (Bashaw and Keister, 2019) as well as maintaining people’s confidence in healthcare organisations.

SSI are related to virulence and microbiological dose, risk factors that can be minimised. Virulence, an organism’s ability to produce toxins or other factors that increase its possibility for survival (Mangram et al., 1999), can be lessened by strategically using antibiotics, but these strategies should ensure that antibiotic resistance is not the end result (Bashaw and Keister, 2019). Similarly, microbiological dose can be lessened by strictly complying with hygienic OR guidelines that maintain low levels of airborne particulates (e.g. ensuring proper ventilation). These practices are essential for keeping the surgical environment as contamination-free as possible. However, as airborne particulates inside an OR are an invisible threat, it is difficult to manage (Qvistgaard et al., 2019). Moreover, managing risks that are not immediately a threat in an OR is not always prioritised in complex or acute care situations.

Covering sterile instruments until they are used is an accepted practice, but no standard exists that identifies how long an unused sterile instrument can be left uncovered. The American Operating Room Association’s (AORN) latest guidelines for sterile technique recommends covering sterile instruments in a manner that allows the cover to be removed without compromising sterility (AORN, 2019). However, sterility is a hypothetical concept that defines a sterile surgical instrument as having a one in a million chance of being contaminated with a live microorganism (Von Woedtke and Kramer, 2008).

AORN encourages the development of standardised procedures for when sterile instruments should be covered and clarification of the length of time they can be covered before use (AORN, 2019). Sweden, where this study was conducted, has no national standard for the covering of sterile instruments before surgery. Local guidelines allow storage of 6–8 h of covered surgical instruments when a surgical procedure is postponed, but the number of covering layers is not specified. These guidelines rely on a study from 2008 (Dalstrom et al., 2008) that found that covered trays with sterile instruments were not contaminated during a 4-h test and found a significant correlation between a tray’s exposure to air and contamination. A pilot study performed in Sweden in 2014 (Sandstrom et al., 2014) also supports the practice of covering sterile instruments when they are not in use.

In order to further examine methods that keep instruments sterile before surgery starts, this study investigates bacterial contamination on set surgical instrument tables with a single- or double-layer drape covering after 15 h. We hypothesised that the double-layer drape would have fewer colony forming units (CFU) than the single-layer drape covering.

## Methods

An expert group, including a professor in microbiology, a biomedical analyst, a nurse specialist in hospital hygiene and an OR nurse, was formed. This group constituted both broad and deep competencies that reflected the study design and ethical aspects. To develop a research protocol, several smaller pilot studies were performed. These pilot studies were used to identify methodological issues and other possible obstacles. For example, one pilot study showed that agar plates dried out and cracked after 24 h with no covering and therefore 24 h was deemed an unsuitable time interval. No agar plates dried out and cracked after 15 h, irrespective of covering.

Ethical aspects of the study design were discussed by the expert group but no ethical approval was sought. According to the Swedish Ethical Review Authority, the requirement for ethical review does not apply to this study.

An experimental design using descriptive and comparative statistics was used. Data collection was conducted from August 2018 to March 2019. Depending on when the research group had access to the OR and competence of an OR nurse, 8–9 instrument tables per time were set up for nine occasions in total. Days between experiments varied from one week to one month within the period of data collection.

## Setting

The experiment was set up in an OR built in 2015. The OR used a ventilation system consisting of a turbulent-mixed airflow with inlet air flow of 700 L/s. Air quality in this type of ventilation is based on dilution. The OR surfaces and floor were cleaned, ventilation was ongoing, the doors to the OR were closed, and no other persons entered the OR while the instrument tables and coverings were arranged. An OR nurse and a circulating nurse set the instrument tables using standard procedures for products (e.g. gowns and drapes) according to SS-EN 13795:2019. Typical sterile surgical instruments such as scissors, tweezers, forceps and needle holders were set on the instrument tables in line with an open surgical procedure, e.g. mastectomy. The sterile instruments used depended on their availability in the OR on the day of the experiment. Contamination of surgical instruments was then indirectly measured by six blood agar plates placed on each instrument table. The Petri dishes used were 9 cm in diameter. After each incubation period, the agar plates were transported to the Clinical Bacteriological Laboratory in Växjö, Sweden where they were incubated at 36 °C for 48 h and the CFU were counted and classified according to species.

## Conduct of experiment

**Table:** 

Step-by-step instructions for conduct of experiment
1. Both OR nurse and circulating nurse entered the OR wearing a clean air suit, a surgical hood and a surgical mask.
2. The OR nurse performed preoperative hand disinfection, gowning and gloving. The circulating nurse performed only hand disinfection.
3. The OR nurse prepared 8–9 instrument tables with sterile drapes to prepare for sterile conditions.
4. The circulating nurse donned sterile gloves and placed six agar plates on each instrument table – one in each corner and two in the middle of the table – and removed the lids.
5. The OR nurse set up the instrument tables (for details, see text).
6. Four instrument tables were covered with a single-layer drape and 30-cm overhang around the table. One corner of the table had an upfolded corner made from the draping. Four instrument tables were covered with a double-layer drape and 30-cm overhang around the table. Only the bottom layer had an upfolded corner.
7. The instrument tables were carefully moved from the OR to a separate section of public area of the hospital for 15 h.
8. After 15 h, instrument tables were carefully moved back to the OR.
9. On the instrument tables with the single-layer drape, the covering was slowly and carefully removed starting at the upfolded corner and using a peeling technique. After 30 s, the lids – stored under a sterile towel – were replaced on the agar plates. The double-layer drapes were removed using the same technique but the bottom layer was removed 30 s after the top layer. Lids were replaced as above.

## Analysis

The non-parametric Wilcoxon signed-rank test was used to determine differences between CFU in the single- and double-layer drape. A *P* value < 0.05 was considered significant. Calculation of power determined the number of agar plates needed to reach significance if the initial trends were continuous. Statistical analyses were conducted using SPSS version 24.

## Results

The experiment consisted of 76 set instrument tables with six agar plates on each table. In total, 36 instrument tables were covered with single-layer draping and 36 instrument tables were covered with double-layer draping. As a control and in parallel with other experiments, one instrument table was set without any covering. Experiments without covering were repeated on four occasions (Table 1).

**Table 1.** Experimental set up and number of CFU on agar plates after 15 h of storage.

Covering	No. of instrument tables	No. of agar plates	Total no. of CFU	Mean CFU per instrument table	Mean CFU per agar plate
No covering	4	24	949	237	39.5
Single-layer drape covering	36	216	142	3.94	0.66
Double-layer drape covering	36	216	75	2.08	0.35

CFU, colony forming units.

**Figure 1.**
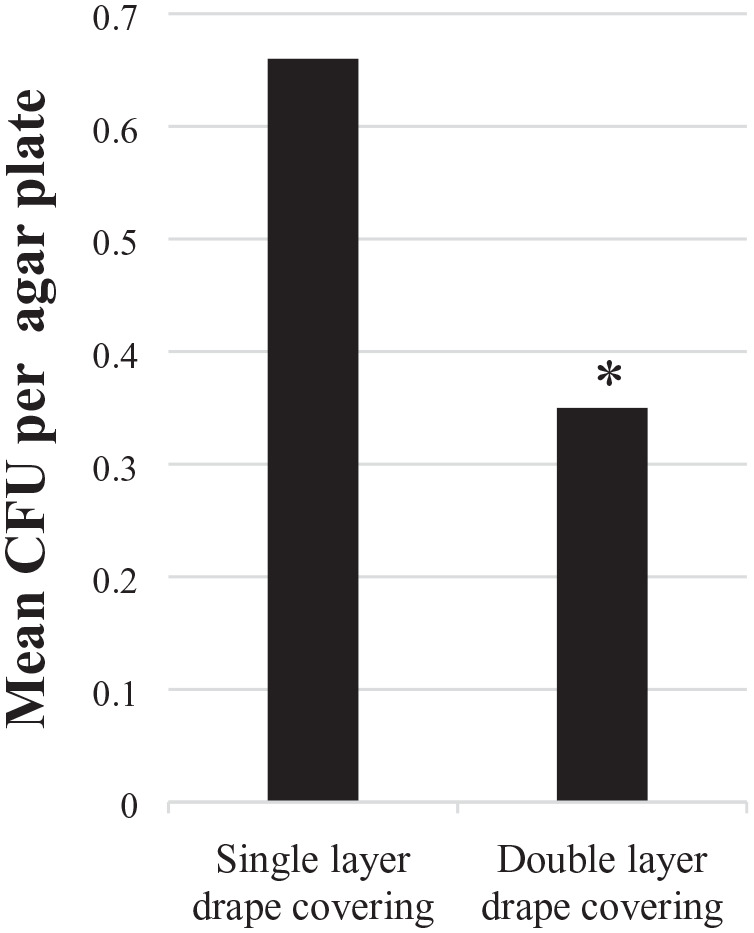
Difference in mean CFU per agar plate for single- and double-layer covering after 15 h of storage. Difference between coverings was significant, *P* = 0.03 (*). CFU, colony forming unit.

After 15 h of storage in an uncontrolled microbiological environment, the double-layer drape covering showed a significantly (*P* = 0.03) lower number of CFU per agar plate than the single layer drape covering (Figure 1).

The uncovered instrument tables were contaminated with a mean of 237 CFU per instrument table (Table 1). As expected, for covered instrument tables, the contamination was much lower.

**Figure 2.**
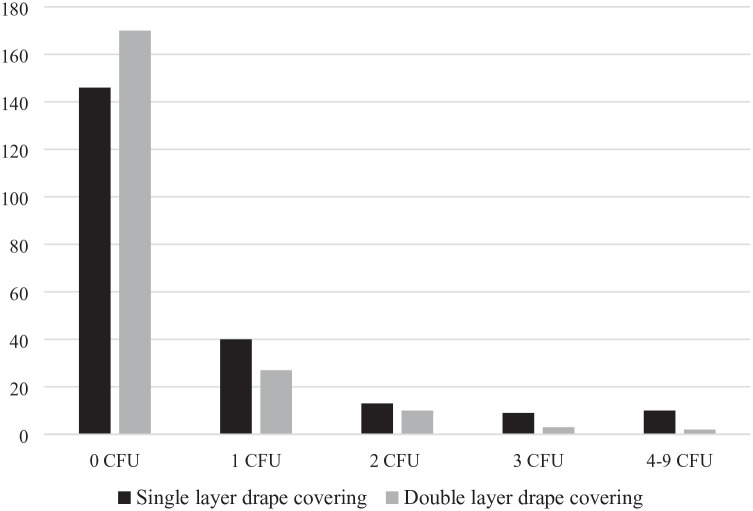
Number of sterile (0 CFU) and non-sterile agar plates with different coverings after 15 h of storage. CFU, colony forming unit.

Out of 432 agar plates with coverings, 319 had no bacterial growth at all (Figure 2), and more agar plates covered with double drapes were sterile than agar plates covered with a single drape. However, this difference was not significant.

## Discussion

The result shows a high amount of sterile agar plates irrespective of covering (single or double layer), which reinforces the importance of restricting exposure to air for sterile surgical instruments pending surgery. The exposure to air is a “danger zone” for sterility and must be controlled by well-implemented hospital hygiene routines and air microbiologically controlled by filtration. Uncovered surgical instruments risk contamination. A previous study also showed that uncovered surgical instruments are at high risk of contamination and should be covered with a sterile towel, which considerably decreases the threat of contamination (Dalstrom et al., 2008).

The difference in number of CFUs was significant between single- and double-layer draping. The advantage of the two-layer principle is that the bottom layer is protected by the top layer. The bottom layer can also be removed with less risks when it has been stored with the protection of a top layer. The surface of the top layer is likely to be as contaminated as instrument tables with no covering so therefore the top layer should be removed before entering the sterile field.

Risk of contamination is highest during set-up and when uncovering the surgical instruments. Setting up surgical instruments in an optimal hygienic environment and then covering them is likely more important than decreasing time between set-up and start of the surgical procedure. After setting the instrument tables, they should be covered with a single drape if they are to be used soon and not moved from the OR. Double draping is needed if the time between set-up and surgery is to be long, i.e. more than 1 h, or if the instrument tables are to be moved. A study from 2017 (Noguchi et al., 2017) shows that unnecessary actions and movements produce a large number of airborne particles and should be avoided near the sterile field. The challenge of maintaining a sterile field in an OR is strongly associated with multiple perioperative personnel interacting (AORN, 2019).

This study has limitations that should be considered. The way of measuring CFU in this study does not directly measure CFU on surgical instruments. The agar plates catch CFU landing on the instrument tables where agar plates are located. CFU landing beside the agar plates are not included. The hypothesis of the method used is that mean contamination of the agar plates is transferable to the remaining area of the instrument table. More than six agar plates on each instrument table makes it impossible to find a place to set up surgical instruments. The number of CFU are not comparable with CFU in cubic metre air, which is a common method of measuring CFU in air samples inside OR. The decision to store the covered instruments in an uncontrolled microbiological environment was carefully thought out and meant to amplify the advantages of the procedure. Still, it is important to emphasise the incorrectness of storing surgical instruments in this type of environment even if they are covered. Therefore, the results should be seen as a trend of numbers of CFU on instrument tables using single and double drape covering.

During the planning of this study, the expert group discussed the different ways to measure CFU on surgical instruments and instrument tables. The method of placing agar plates on instrument tables and counting the CFU has been used in earlier studies (Hughes, 2013). Using a multidisciplinary expert group in the beginning of a project is an important factor for a well-designed study (Dawson, 2019). The expert group working on this project has been an inestimable support throughout the study.

The most important clinical impact of this study is determining the optimal way of storing surgical instruments pending surgery. Results indicate the importance of optimal conditions when exposing the surgical instruments to air. Our results indicate that it is good practice to cover instruments properly with at least a single-layer drape before a surgical procedure. If there is difficulty achieving optimal conditions while setting the instrument tables (e.g. positioning the patient for general anaesthesia), it is a better option to set the instrument tables earlier and cover them with a double layer drape. These precautions will help protect the patient from harm and unnecessary SSI by lowering microbiological burden, a key factor in developing SSI. Surgery patients are vulnerable and need to be thoroughly cared for. SSI can be reduced in the long term using multidisciplinary care bundles (Weiser et al., 2018), including the proper use of drapes over surgical instruments. Lastly, this study exemplifies effective use of OR competence, suggesting a procedure of setting the surgical instruments when the OR nurse has opportunity and optimal conditions for this important assignment.
